# Relationships of Alpha-SMA-Positive Fibroblasts and SDF-1-Positive Tumor Cells with Neoangiogenesis in Nasopharyngeal Carcinoma

**DOI:** 10.1155/2014/507353

**Published:** 2014-04-27

**Authors:** Shumin Wang, Ning Ma, Shosuke Kawanishi, Yusuke Hiraku, Shinji Oikawa, Ying Xie, Zhe Zhang, Guangwu Huang, Mariko Murata

**Affiliations:** ^1^Department of Environmental and Molecular Medicine, Mie University Graduate School of Medicine, 2-174 Edobashi, Tsu, Mie 514-8507, Japan; ^2^Department of Otolaryngology Head and Neck Surgery, First Affiliated Hospital of Guangxi Medical University, No. 22 Shuangyong Road, Nanning 530027, China; ^3^Faculty of Nursing Science, Suzuka University of Medical Science, 3500-3 Minamitamagaki-cho, Suzuka, Mie 513-8670, Japan; ^4^Faculty of Pharmaceutical Sciences, Suzuka University of Medical Science, 3500-3 Minamitamagaki-cho, Suzuka, Mie 513-8670, Japan

## Abstract

Nasopharyngeal carcinoma (NPC) is one of the most prevalent malignant tumors with poor prognosis in Southern China and Southeast Asia. Angiogenesis-related molecules can be promising therapeutic targets in NPC. To investigate the relationships of cancer-associated fibroblasts (CAFs) and chemokine-related molecules with neoangiogenesis, we compared immunohistochemical analyses of alpha-smooth-muscle actin (**α**-SMA), stroma-derived factor-1 (SDF-1), and its receptor CXCR4 in primary NPC specimens and chronic nasopharyngitis tissues. In addition, we examined the expression of vascular endothelial growth factor (VEGF-A), and CD133- and VEGF- receptor-2 (VEGFR-2) double positive cells, as endothelial progenitor cells (EPCs). We also assessed CD34-positive microvessels. Significantly higher expression of **α**-SMA was observed in fibroblasts in NPC stroma. The immunoreactive intensities of SDF-1 and CXCR4 were significantly higher in NPC cells. CXCR4-positive cells and CD133/VEGFR-2- double positive cells were observed in the stroma surrounding cancer nests, and VEGF was detected in both cancer and stromal cells. Microvessel density was significantly higher in the stroma of NPC tissues compared to chronic nasopharyngitis tissues. Our data suggest that CAFs and NPC tumor cells may enhance neoangiogenesis in a VEGF- and SDF-1-dependent manner by recruiting EPCs from the bone marrow into tumor stroma.

## 1. Introduction

Genetic and cell biology studies indicate that tumor growth is determined not only by malignant cancer cells but also by the tumor stroma [1]. Fibroblasts in the tumor stroma acquire a perpetually activated phenotype and become a subpopulation that can be identified by the expression of *α*-smooth-muscle actin (*α*-SMA) [2]. *α*-SMA-positive fibroblasts are called cancer-associated fibroblasts (CAFs) [2]. Many reports have indicated that CAFs are important promoters of tumor growth and progression, as their production of growth factors and chemokines and extracellular matrix facilitate the angiogenic recruitment of endothelial cells and pericytes [2–4].

Stroma-derived factor-1 (SDF-1) is an *α*-chemokine that binds only to the receptor CXCR4. Recent studies have suggested that the SDF-1/CXCR4 axis plays an important and unique role in the egress of hematopoietic stem/progenitor cells from bone marrow [5, 6]. Hematopoietic stem/progenitor cells are recruited into the cancer stroma, where they become endothelial progenitor cells (EPCs). CD133 is a hematopoietic stem/progenitor cell marker [7] and unexpressed on mature differentiated endothelial cells [8]. Vascular endothelial growth factor receptor-2 (VEGFR-2), also known as Flk1, is an early endothelial marker [9]. CD34 also includes endothelial antigens [10]. The double positive cells of CD133 with VEGFR-2 or CD34 are verified as EPCs [8], which may contribute to angiogenesis and tumor development [11].

Microvessel density measurement is a quantitative method of assessing angiogenesis [12]. CD34 can be used as an indicator of microvessel density [10]. Tumor microvessel density is correlated with the concentration and expression of proangiogenic growth factors such as vascular endothelial growth factor (VEGF) [13]. VEGF-A, a 34- to 46-kDa glycoprotein, is a potent stimulator of endothelial cell growth, and it can stimulate both physiological and pathological angiogeneses [14].

Nasopharyngeal carcinoma (NPC) is one of the most prevalent malignant tumors in Southern China and Southeast Asia, and its prognosis has been poor for decades [15]. Therefore, a better understanding of its pathogenesis is needed. Tumor progression clearly depends on angiogenesis [16], and, thus, targeting tumor angiogenesis is a promising strategy for NPC and many solid tumors [17]. To investigate the relationships of cancer-associated fibroblasts (CAFs) and related molecules to neoangiogenesis, we performed immunohistochemical analyses of alpha-smooth-muscle actin (*α*-SMA), stroma-derived factor-1 (SDF-1), and its cognate receptor CXCR4 in primary NPC lesions. We also examined EPCs by double fluorescent staining of CD133 with VEGFR-2 and CD34. In addition, to evaluate neoangiogenesis, we observed microvessels by CD34 staining and their growth factor VEGF-A.

## 2. Material and Methods

### 2.1. Patients

Formalin-fixed and paraffin-embedded biopsies were obtained from 57 patients (45.2 ± 10.7 years old, 38 males and 19 females) with NPC, and chronic nasopharyngitis tissues were obtained via tonsillectomy from 31 patients (40.1 ± 11.9 years old, 20 males and 11 females) with chronic nasopharyngitis, who functioned as normal controls. All subjects were patients at the Department of Otolaryngology-Head and Neck Surgery, First Affiliated Hospital of Guangxi Medical University, Nanning, China, and provided informed consent prior to participation. Diagnoses were made by experienced pathologists according to the World Health Organization (WHO) classification. The pathological diagnosis of all NPC samples was nonkeratinizing carcinoma. This study was performed in accordance with ethical review committee approval notice (2009-07-07) of the First Affiliated Hospital of Guangxi Medical University, China, and ethical approval (number 1116) by Mie University, Japan. We removed identifying information from all samples before analysis.

### 2.2. Immunoperoxidase Study of *α*-SMA, CXCR4, VEGF-A, and CD34

Standard immunoperoxidase methods were used to examine the distribution of *α*-SMA, CXCR4, VEGF-A, and CD34 in NPC tissues and normal controls. After deparaffinization and rehydration, antigen was retrieved in 5% urea buffer by microwave heating for 5 min and then incubated in 1% H_2_O_2_ for 30 min to block endogenous peroxidase activity. Sections of 3 *μ*m thickness were incubated overnight at room temperature with the following antibodies: rabbit polyclonal anti-*α*-SMA (1 : 200, Abcam, Cambridge, MA), rabbit monoclonal anti-CXCR4 (1 : 100, Abcam), rabbit polyclonal anti-VEGF (human VEGF-A165, 1 : 200, Abcam), and mouse monoclonal anti-CD34 (1 : 200, Monosan, Uden, Netherlands). For the rabbit antibodies (*α*-SMA, CXCR4, and VEGF-A), the sections were incubated with goat anti-rabbit IgG for 3 h, then incubated with peroxidase antiperoxidase complex for 2 h. For mouse antibody (CD34), the sections were incubated with biotinylated anti-mouse IgG for 3 h then incubated with avidin-biotin complex (Vectastain ABC kit, Vector Laboratories, Burlingame, CA) for 2 h. Sections were then incubated with 3,3′-diaminobenzidine (DAB substrate kit; Vector Laboratories). Nuclei were counterstained by hematoxylin.

### 2.3. Immunofluorescence Study of SDF-1, CD133, VEGFR-2, and CD34

Immunoreactivities of SDF-1 in nasopharyngeal tissues were assessed by single immunofluorescence labeling study. Double positive cells of CD133 with VEGFR-2 or CD34 were detected by double immunofluorescence labeling studies. Briefly, deparaffinized and rehydrated sections (3-*μ*m thickness) were incubated with 5% skim milk and were then incubated with mouse monoclonal anti-SDF-1 (1 : 100, Santa Cruz Biotechnology, CA), mouse monoclonal anti-CD133 (1 : 100, Abgent Inc., San Diego, CA), rabbit monoclonal anti-CD133 (1 : 100, Abcam), rabbit polyclonal anti-VEGFR-2 (1 : 50, Abcam), or mouse monoclonal anti-CD34 (1 : 200, Monosan) as the primary antibody overnight at room temperature. The sections were then incubated for 3 h with Alexa 594-labeled goat antibody against rabbit IgG or Alexa 488-labeled goat antibody against mouse IgG (1 : 400) (Molecular Probes, Eugene, OR). Stained sections were examined using fluorescence microscopy (BX53, Olympus, Tokyo, Japan).

### 2.4. Immunohistochemical Grading

Immunohistochemical (IHC) grading based on intensity and frequency of staining results was performed by two independent investigators without knowledge of the patients' clinicopathological features. The staining intensity was scored as negative (0), weak (+1), moderate (+2), or strong (+3). Frequency of positive cells in specific areas was scored as negative (0), less than 25% (+1), 25–50% (+2), 51–75% (+3), or more than 75% (+4). IHC grades were assigned by multiplying the intensity score by the frequency score as follows: −, absent expression (0); +, weak expression (1); ++, moderate expression (2); +++, high expression (3); or ++++, very high expression (4).

### 2.5. Microvessel Evaluation

Microvessel density (MVD) was estimated using a light microscope. We checked MDV by two methods; one was counting number of microvessels and the other was digital microscopy assessment. Prior to counting the microvessels, slides were examined at low power magnification (×40) to identify the area with the highest density of the microvessels. The five most vascular fields within the section were selected, and MVD measurement was performed at high power magnification (×200). For counting microvessels, areas staining for CD34, whether single endothelial cells or clusters of endothelial cells, regardless of the absence/presence of a lumen were counted as individual microvessels. The microvessel numbers of the five most vascular areas were averaged to give an estimate of tissue microvessel density for each patient. For digital microscopy assessment of MVD, the intensity and area of endothelial staining were quantitatively measured using the cellSens Standard Ver1.4 Imaging Software (Olympus, Tokyo, Japan). The immunohistochemical stain was then selected using the “color selection” function and the “area/density (intensity) measurement” functions were used to calculate the respective values.

### 2.6. Statistical Analysis

Statistical differences were determined by the chi-square test and Mann-Whitney *U* test. *P* < 0.05 was considered to be statistically significant. Correlation between factors was assessed with the Spearman correlation test. Statistical analysis was performed using SPSS19 for Windows.

## 3. Results

### 3.1. Expression of *α*-SMA in the Stroma of NPC Tissues

**Figure 1:**
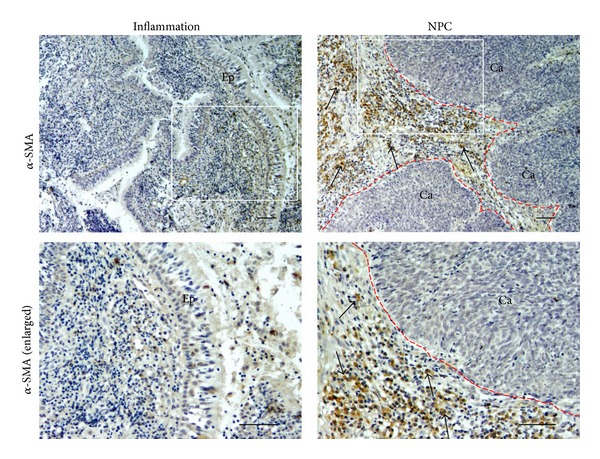
The expression of *α*-SMA in nasopharyngeal tissues. Formalin-fixed and paraffin-embedded biopsies of nasopharyngeal tissues were obtained from chronic nasopharyngitis (inflammation) and NPC tissues. The expression of *α*-SMA was assessed by immunoperoxidase staining (brown). Arrows indicate *α*-SMA-positive cells. The red dotted line is border between cancer nest and stromal area. Original magnification is 100x (upper) and the insets are enlarged in the lower panels (200x). Scale bar represents 50 *μ*m. Ep: epithelium. Ca: cancer cells.

Figure 1 shows the localization of *α*-SMA in nasopharyngeal tissues from patients with NPC and chronic nasopharyngitis (inflammation). In the chronic nasopharyngitis samples, *α*-SMA expression was scarce and was detected only in vascular pericytes and vascular smooth muscle (data not shown). No immunoreactivity was observed in normal nasopharyngeal epithelium or stromal fibroblasts. In contrast, tumor cells showed negative immunoreactivity for *α*-SMA, but *α*-SMA-positive stromal cells (arrows) were observed surrounding cancer nests. *α*-SMA was expressed in the cytoplasm of fibroblasts in primary NPC tissues. In most of the NPC cases, a large proportion of fibroblasts were *α*-SMA positive.

### 3.2. Expression of SDF-1 and Its Receptor CXCR4 in Nasopharyngeal Tissues

**Figure 2:**
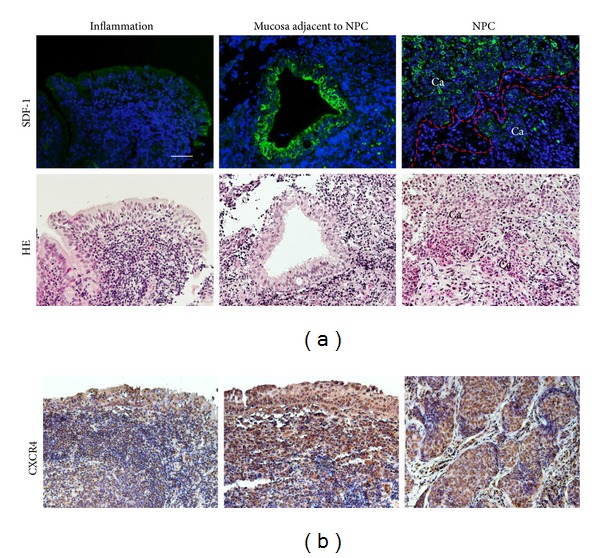
The expression of SDF-1 and CXCR4 in chronic nasopharyngitis (inflammation) and NPC tissues. (a) The expression of SDF-1 was assessed by immunofluorescence staining (green). Nuclei were counterstained by DAPI (blue). The red dotted line is border between cancer nest and stromal area. HE staining of parallel sections. (b) Immunoperoxidase staining of CXCR4 protein (brown). Original magnification is 200x. Scale bar represents 50 *μ*m. Ca: cancer cells.

Figure 2 shows the expression patterns of cancer-specific cytokine SDF-1 and its receptor CXCR4 in primary NPC and chronic nasopharyngitis tissues. In chronic nasopharyngitis tissues (inflammation), epithelial cells showed weak SDF-1 and CXCR4 immunoreactivity (Figures 2(a) and 2(b), left). In primary NPC tissues, SDF-1 was intensively expressed in the cytoplasm and membrane of NPC tumor cells (Figure 2(a), right) and mucosal epithelial cells adjacent to NPC nest (Figure 2(a), middle) but not in the stromal cells (Figure 2(a), right). CXCR4 was strongly expressed in the nucleus, cytoplasm, and cellular membrane of NPC cells and mucosa adjacent to NPC nest (Figure 2(b), middle and right).

### 3.3. Expression of CD133 and VEGF-A in NPC Tissues

**Figure 3:**
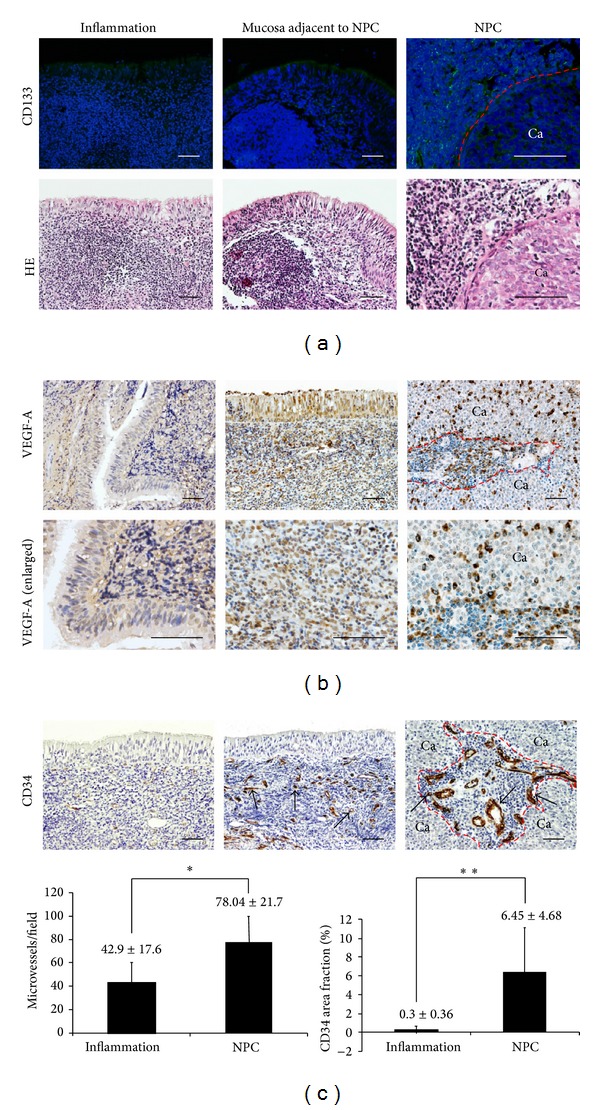
Staining patterns of CD133, VEGF-A, CD34, and microvessel density in chronic nasopharyngitis (inflammation) and NPC tissues. (a) The expression of CD133 by immunofluorescence staining (green). Nuclei were counterstained by DAPI (blue). HE staining of parallel sections. Immunoperoxidase staining of (b) VEGF-A (brown) and (c) CD34 (brown). Arrows show CD34-positive vessels. The red dotted line is border between cancer nest and stromal area. Original magnification is 200x (VEGF-A in upper panels, CD34) and VEGF-A enlarged in the lower panels (400x). Scale bar represents 50 *μ*m. Ca: cancer cells. Graphs represent average and SD of microvessels/field and CD34 area fraction (%) for inflammation (*n* = 6) and NPC tissues (*n* = 10). *P* values were calculated by the Mann-Whitney *U* test in comparison to chronic nasopharyngitis tissues. *P < 0.05; **P < 0.01.

Figure 3 shows the expression patterns of angiogenesis-related molecules in primary NPC and inflammatory tissues. Abundant CD133-positive cells were observed in the stroma of NPC (Figure 3(a), right) but not in nasopharyngitis tissues (Figure 3(a), left). Weak VEGF-A immunoreactivity was observed in the epithelial cells and stromal cells of inflammatory tissues (Figure 3(b), left). On the other hand, a predominantly membrane/cytoplasmic distribution was seen in both stromal cells and NPC cells (Figure 3(b), middle and right).

### 3.4. CD34-Positive Microvessels and Evaluation of MVD in NPC Tissues

Figure 3(c) shows the membrane and cytoplasm of vascular endothelial cells were stained with CD34 in the stroma of nasopharyngeal tissues. The microvessels in section labeled by anti-CD34 presented single endothelial cell or small clusters of the cells, with or without an irregular lumen (Figure 3(c), arrows). CD34-positive vessels were more abundant in the stromal tissue of NPC samples (Figure 3(c), middle and right) than in inflammatory tissues (Figure 3(c), left), indicating higher formation of microvessels in NPC. Distribution of the microvessels within the tumor was uneven and heterogeneous. The area of highest vascularization was usually in the stroma between the NPC nests. CD34-positive cell clusters were counted as microvessel density at high power magnification (×200). Number of microvessels/field was significantly higher in NPC stroma (78.04 ± 21.7) than inflammatory tissues (42.9 ± 17.6, *P* < 0.05). CD34-positive area fraction (%) was significantly higher in NPC (6.45 ± 4.68) than inflammation (0.3 ± 0.36, *P* < 0.01).

### 3.5. Expression of CD133 with VEGFR-2 or CD34 in Stromal Cells

**Figure 4:**
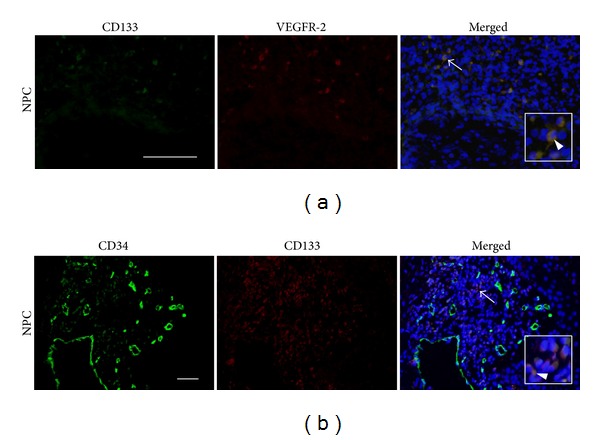
Double immunofluorescent staining of CD133 with VEGFR-2 and CD34 in NPC tissues. (a) The immunofluorescence expression of CD133 (green) and VEGFR-2 (red). (b) The immunofluorescence expression of CD34 (green) and CD133 (red). Nuclei were counterstained by DAPI (blue) in the merged pictures. Arrows and arrowheads indicate the double positive cells and the enlarged (insets). Original magnification is 400x (a) and 200x (b). Scale bar represents 50 *μ*m.

Figure 4 shows stromal cells with CD133, VEGFR-2 and CD34 immunoreactivities in NPC tissues. Double immunofluorescent staining of the stem/progenitor cell marker and endothelial marker showed a small number of CD133 and VEGFR-2 double positive cells in the stroma surrounding NPC nests (Figure 4(a), merged, arrow, enlarged picture in inset, and arrowhead). We also observed double immunostaining of CD133 and CD34 in membrane and cytoplasm of some cells in the stroma (Figure 4(b), merged, arrow, enlarged picture in inset, and arrowhead).

### 3.6. Relationship of *α*-SMA and Related Molecules in Nasopharyngeal Samples

Table 1 shows statistical analyses of the expression levels of *α*-SMA and angiogenesis-related molecules with comparisons between NPC and chronic nasopharyngitis patients. The immunoreactivity of *α*-SMA was significantly increased in NPC tissues relative to inflammatory tissues (*P* < 0.001). CD133, VEGFR-2, and CD34 also showed significantly higher expression in the stroma of primary NPC tissues compared with inflammatory tissues (CD133, *P* = 0.006; VEGFR-2, *P* = 0.04; CD34, *P* < 0.001). CXCR4 and VEGF-A were observed in both stromal and cancer cells, and the IHC grades were significantly higher in NPC than chronic nasopharyngeal tissues (CXCR4, *P* = 0.041; VEGF-A, *P* = 0.015). SDF-1 was highly expressed in cancer cells of NPC tissues than inflamed tissues (SDF-1, *P* = 0.008). We found a significant correlation between *α*-SMA and CD34 (Spearman rank correlation coefficient; *r* = 0.721, *P* < 0.001) in nasopharyngeal tissues. SDF-1 expression was also positively correlated with CD34 expression (*r* = 0.701, *P* = 0.002).

## 4. Discussion

In this study, we firstly found *α*-SMA-positive fibroblasts in the stroma of NPC tissues. CAFs, activated fibroblasts that often express *α*-SMA within desmoplastic lesions, are associated with cancer progression [2]. Their involvement has been reported in tumorigenesis and metastasis in several types of cancers [3, 18–21]. *α*-SMA was shown to be useful for predicting patient prognosis of pancreatic ductal adenocarcinoma [18]. In our study, we demonstrated that a large proportion of stromal cells surrounding cancer nests were *α*-SMA positive in most of the NPC samples but not in the chronic nasopharyngitis samples, suggesting the involvement of CAFs in NPC progression.

Several studies showed high expression of SDF-1/CXCR4 in many cancers [4, 22–24], including NPC [25, 26]. In this study, SDF-1 and its receptor CXCR4 were intensively expressed in primary NPC samples. SDF-1 was strongly expressed in NPC tumor cells and mucosal epithelial cells adjacent to NPC nest, but it was not expressed in stromal cells, including CAFs. Although SDF-1 is known to be released by CAFs in several cancers [3, 27, 28], SDF-1 could be produced by cancer cells as a paracrine factor [24]. High expression of SDF-1 of tumor cells forms a local gradient of the chemokine in the tumor region, recruiting CXCR4-expressing bone marrow-derived progenitor cells to the tumor [11]. Our results suggested that NPC tumor cells secrete SDF-1 into the stroma and cancer cells themselves in paracrine and autocrine loops, and this chemokine may contribute to the process of vasculogenesis by recruiting CXCR4-expressing cells, such as inflammatory cells and endothelial progenitor cells, to the NPC mass.

We observed strong expression of VEGF-A in both stromal cells and tumor cells of NPC tissues, indicating that stromal cells and NPC cells can secrete significant amounts of VEGF. VEGF is believed to play an important role in angiogenesis. It is responsible for initiating capillary growth by attracting and stimulating endothelial cells in the reactive stroma [29]. Fibroblasts and inflammatory cells are the principal source of host-derived VEGF [30]. Cancer cells themselves can also release VEGF [31–33]. Critical steps in tumor angiogenesis include the recruitment of circulating endothelial progenitor cells [34] and vascular endothelial cell migration [35]. CAFs enhance tumor angiogenesis by inducing mobilization and recruitment of circulating endothelial progenitor cells [2, 3]. Such endothelial progenitor cells likely enter the tumor stroma from the bone marrow via the peripheral circulation [36], and the recruitment is mediated in part by SDF-1 and VEGF [36]. Kryczek et al. suggested that tumor-derived SDF-1 and VEGF could synergize to stimulate vascular endothelial cell proliferation and protect against cell apoptosis [4]. Therefore, both SDF-1 and VEGF could contribute to angiogenesis in NPC.

Peichev et al. [8] found that incubation of CD133^+^VEGEF-2^+^ cells, putative EPCs, with VEGF and other factors resulted in differentiation into mature CD133^−^VEGEF-2^+^ cells. Hwang et al. [37] characterized EPCs and endothelial cells by RT-PCR, and EPCs were positive for cell markers CD133 and CD34, and after differentiation into endothelial cells, they highly expressed CD34 but not CD133. Interestingly, in our study, double positive cells of CD133 with VEGFR-2 or CD34 were observed surrounding NPC nests, which may be consistent with the hypothesis [38] regarding circulating endothelial progenitor cells' mobilization and migration. Tumor angiogenesis is indispensable to tumor growth, invasion, and metastasis, and the current gold standard to characterize tumor angiogenesis is histological MVD technique [39]. Microvessel density is an outcome indicator in several malignancies [10, 40–42], including NPC [43]. In the present study, high microvessel density was obtained in NPC stroma and putative EPCs were observed near microvessels, suggesting the tumor-associated neoangiogenesis in NPC. Since concomitant chemoradiotherapy (i.e., cisplatin and 5-fluorouracil followed by radiation) is currently recommended for invasive NPC [44], antiangiogenic therapy in combination with cytotoxic therapies [45] would help to improve general treatment approaches for NPC. Our present study provides the possibility of targeting angiogenesis-related molecules and CAFs for treating NPC. Further studies are required for elucidating the mechanism of facilitating angiogenesis in nasopharyngeal carcinoma.

## 5. Conclusions

Our observations suggested that *α*-SMA-positive fibroblasts (CAFs) and NPC tumor cells enhanced neoangiogenesis in a VEGF- and SDF-1-dependent manner.

## Figures and Tables

**Table 1:** Expression of *α*-SMA and related molecules in nasopharyngeal tissues.

	Location		Group	IHC grade^#^					*P* value*
	Stroma	Cancer nest		−	+	++	+++	++++	
*α*-SMA	Positive^$^	ND	Chronic nasopharyngitis (21)	11	7	2	1	0	0.000
			NPC (18)	0	4	0	7	7	

CD133	Positive	ND	Chronic nasopharyngitis (10)	9	1	0	0	0	0.006
			NPC (30)	8	9	9	4	0	

VEGFR-2	Positive	ND	Chronic nasopharyngitis (8)	7	1	0	0	0	0.04
			NPC (10)	2	5	1	2	0	

CD34	Positive	ND	Chronic nasopharyngitis (14)	0	8	6	0	0	0.000
			NPC (24)	0	0	3	7	14	

CXCR4	Positive	Positive	Chronic nasopharyngitis (6)	0	3	1	2	0	0.041
			NPC (11)	0	0	2	4	5	

VEGF-A	Positive	Positive	Chronic nasopharyngitis (10)	0	1	5	4	0	0.015
			NPC (19)	0	0	2	9	8	

SDF-1	ND	Positive	Chronic nasopharyngitis (22)	5	9	5	2	1	0.008
			NPC (20)	3	0	7	5	5	

^#^IHC grades were assigned to each specimen according to the grade of staining intensity as described in Section 2.

*P values were calculated by the chi-square test in comparison to chronic nasopharyngitis tissues.

^$^IHC reactivity: positive; ND: not detected.

## Abbreviations

NPC: Nasopharyngeal carcinoma
CAF: Cancer-associated fibroblast
*α*-SMA: Alpha-smooth-muscle actin
SDF-1: Stroma-derived factor-1
VEGF: Vascular endothelial growth factor
EPC: Endothelial progenitor cell
MVD: Microvessel density
IHC: Immunohistochemistry.
